# URCHOICE: Preferences for Pre-Exposure Prophylaxis (PrEP) Options for HIV Prevention Among Kenyan men who have sex with men and Transgender Women in Nairobi, Kisumu and the Coast

**DOI:** 10.1007/s10461-022-03741-2

**Published:** 2022-06-10

**Authors:** Robert C. Bailey, Makobu Kimani, Rhoda Kabuti, Edwin Gumbe, George Otieno, Joshua Kimani, Duncan Okall, Eduard J. Sanders, Fredrick O. Otieno

**Affiliations:** 1 Division of Epidemiology and Biostatistics, School of Public Health, University of Illinois at Chicago, 1603 West Taylor Street, 60612 Chicago, IL, USA; 2 Nyanza Reproductive Health Society, Kisumu, Kenya; 3 KEMRI/Wellcome Trust Research Programme Centre for Geographic Medicine Research, Kilifi, Kenya; 4 Partners for Health and Development in Africa, Nairobi, Kenya; 5 Medical Research Institute Center for Microbiology Study, Research Care & Training Programme, Kisumu, Kenya; 6 Department of Global Health, University of Amsterdam, Amsterdam, The Netherlands; 7 Nuffield Department of Medicine, University of Oxford, Headington, United Kingdom

**Keywords:** HIV, Men who have sex with men, Transgender Women, Pre-exposure Prophylaxis, Long-acting PrEP, Injectables, Implants

## Abstract

HIV prevention method preferences were evaluated among Kenyan men who have sex with men (MSM) and transgender women (TW) from three sites: Kisumu, Nairobi and the Coast. Information sessions detailing the attributes, duration of protection, route of administration and probable visibility were attended by 464 HIV negative participants, of whom 423 (median age: 24 years) agreed to be interviewed. Across pairwise comparisons daily PrEP was by far the least preferred (1%); quarterly injections (26%) and monthly pills (23%) were most preferred, followed by yearly implant (19%) and condoms (12%). When participants were “forced” to choose their most preferred PrEP option, only 10 (2.4%) chose the daily pill; more (37.1%) chose the quarterly injection than the monthly pill (34.8%) and the yearly implant (25.8%). TW preferred the yearly implant over the quarterly injection. To achieve the rates of PrEP uptake and adherence necessary for protecting large proportions of vulnerable MSM and TW, a variety of long-acting products should be developed and made accessible to appeal to a diversity of preferences.

## Introduction

The HIV pandemic has disproportionally impacted gay, bisexual, and other men who have sex with men (MSM) and transgender women (TW) globally. Prevention of new infections in these populations is of paramount importance [1–3]. Daily pre-exposure prophylaxis (PrEP) with tenofovir disoproxil fumarate–emtricitabine (TDF–FTC) has been shown to reduce risk of HIV acquisition among MSM and TW by 44% [4], with up to 90% effectiveness among participants who maintain high levels of intracellular tenofovir diphosphate (TFV-DP) as seen in peripheral blood mononuclear cells [5]. While daily or near daily PrEP unquestionably has the potential to reduce new HIV infections, post-trial analyses have shown repeatedly that PrEP efficacy depends on adherence as measured by drug detection in serum or tissues [5, 6]. For many, adherence to a daily regimen has proven difficult.

In Kenya, PrEP was officially adopted in 2017 as part of combination HIV prevention for those at substantial ongoing risk, including MSM and TW [7]. Since then, several studies in that country have shown that, despite high levels of self-reported adherence, levels of drug found in serum of MSM and TW were very low and insufficient to achieve protection. For example, one small Kenyan trial comparing daily to intermittent PrEP enrolled 62 MSM and demonstrated high acceptability [8, 9]. Median adherence to daily PrEP, using electronic monitoring, was 80% of prescribed doses [10]. However, an assessment of PrEP adherence at the same research clinic, showed that among 76 MSM who reported PrEP use, only 11 (14.5%) had protective TFV-DP levels (≥ 4 dose per week) [11]. Another small study on the Coast of Kenya found that, among 53 MSM and TW participants who initiated PrEP, just 3 of 11 TW and no MSM had protective levels of TFV-DP after six months [12]. Similarly, in an observational study conducted in Kisumu, Kenya, among 157 MSM initiating PrEP, visual analog scale ratings of past-month adherence were very high - ranging from 8 to 100%, with a median of 98% (interquartile range, 90–100%). Yet any TFV-DP was detected at months 3 and 9 of follow-up in only 73 (46.5%) individuals, with protective levels in just 23 (14.6%) individuals [13].

Effective alternatives to once daily oral PrEP are urgently needed. As new longer-acting formulations and delivery options are developed and tested, it will be prudent to understand the preferences of the intended end users for different prevention modalities. A new product may have the potential to reduce adherence challenges, be used discreetly and ease some health systems challenges, but these benefits will only materialize if people prefer and use the product. While there have been studies of preferences for different formulations among MSM in the U.S. [14–21], studies of preferences among MSM and TW in sub-Saharan Africa have been limited to one multinational study assessing preferences between oral and injected PrEP [22] and one discrete choice experiment assessing various attributes of daily versus longer acting PrEP among youth in South Africa [23]. End-user input on early-stage design of new HIV prevention approaches is critical to yielding products that achieve high uptake and adherence.

The aims of this cross-sectional study conducted at three sites in Kenya were to (a) evaluate preferences for current and future HIV prevention options; and (b) to assess socio-demographic and behavioral factors associated with each preference. Five different HIV prevention methods were evaluated in pairwise comparisons: condoms, daily oral pill, monthly pill, three-monthly injection, and yearly implant, resulting in ten pairwise comparisons.

## Methods

### Study Sites and Participant Recruitment

The study was conducted at three sites in Kenya: Kisumu, Nairobi and the Coast. The Coast included participants from both Malindi and Mombasa. Participants were recruited from clients of local MSM and TW support organizations and from those participating in HIV prevention programs through partner organizations and at county health facilities. Individuals who reported to be HIV negative were invited to attend an information session that lasted 90 min to two hours and included information about different methods of preventing HIV transmission. All participants provided signed informed consent prior to each information session. The prevention methods covered included condom use, daily oral PrEP, intermittent or event-driven PrEP, a monthly pill, a three-monthly (quarterly) injection, and a once per year (yearly) implant. Each description highlighted key attributes of each method including method and frequency of administration, duration of efficacy, and perceptibility. Participants were told that the PrEP regimens were all equally highly effective if taken properly and consistently. Sources of information were taken from the literature (condoms, daily oral and intermittent PrEP), from information provided by Merck Sharpe and Dhome, the sponsors of the study whose products are in development (monthly pill, three-monthly injection and yearly implant), and from study team’s expertise in the fields of epidemiology, HIV prevention and sexual and reproductive health. Attendance at information sessions ranged from 10 to 40 participants, depending on Covid guidelines at any location. After each session, participants were invited to volunteer and be scheduled for an individual interview.

To be eligible to participate in an interview, the volunteer had to be 18–35 years of age, male at birth, agree to be tested for HIV, test HIV negative by rapid test, and be able to provide informed consent. HIV tests were performed by trained counselors certified by the Kenya National AIDS and STI Control Program using Determine™ HIV-1/2 Ag/Ab Combo according to Kenya Ministry of Health guidelines [24]. All the study sites were attached to facilities that provided HIV prevention, care and treatment services tailored for MSM and TW. Just one of the volunteers tested positive and was referred to care on the same day.

### Interviews

Face-to-face interviews took place in a private setting in the language of the participant’s choosing (English, KiSwahili, DhoLuo). Interviewers were peers who had assisted with the information sessions and had previous interviewing experience. The interview questionnaire covered demographic variables, sexual orientation, sexual behaviors, and PrEP awareness and use. Participants were then presented with a series of randomized pairwise choices among the five options (condoms, daily pill, monthly pill, quarterly injection, yearly implant) and asked to select their preferred option. All 10 possible pairwise combinations of the five options (e.g., condoms vs. daily pill, condom vs. monthly pill, condom vs. quarterly injection, etc.) were included. Participants were then “forced” to choose the one option they preferred most. While this was a primarily quantitative study, to complement the quantitative data and to gain insight into the reasons for participants’ choices, we asked for brief responses to the qualitative question, “What are the reasons for making the choice that you did?”

### Data Management and Analyses

Responses were entered on a handheld tablet using QDS Computer Assisted Personal Interview (CAPI) and the data were transferred to STATA version 16.1 (STATA Corporation, College Station, Texas, USA) for analyses. Variables were summarized using frequency counts and percentages and stratified by paired preference choices. A preference was assigned to one particular prevention method if that one method was consistently selected over each of the other options in all possible pairwise comparisons. If participants did not consistently choose one option over the other four in all pairwise comparisons, then “Undecided” was assigned [16]. Distributions were compared by whether the respondent had a preferred method of HIV prevention or was Undecided, using non-parametric Kruskal-Wallis Tests. If there was at least one significant difference in the overall test, Dunn’s Pairwise (post hoc type) Test analyses were conducted to determine which groups(s) outperformed which. The frequencies and percentages of the forced choices were calculated and a multinomial regression was generated to assess the relative risk ratios (RRR) of demographic and behavioral factors associated with choice of monthly pill or quarterly injection compared to the yearly implant. Daily oral pill was excluded because of so few respondents expressing preference for that option. Factors that were significant at p < 0.10 were then retained in a subsequent multinomial regression model to arrive at adjusted RRRs of monthly pill and quarterly injection compared to yearly implant. We chose p < 0.10 as the stopping point as p-values are driven by sample size, and many variables 0.05 < p < 0.10 have large effect sizes. Therefore, we lean to the side of sensitivity to identify factors that may be of importance to consider when these theoretical findings are translated to implementation.

### Qualitative Analyses

All participants responded to the question asking for the reasons that they made the final choice that they did. The responses within each choice were listed and then grouped by themes that emerged for each choice. These being qualitative responses, frequencies of the different themes are not presented as they would suggest a precision not possible given the variation in how concepts were expressed and the necessary subjectivity involved in grouping. We report which themes were most salient and indicate approximate proportions of participants giving different responses by applying the following terms: “a few” (approximately 1-10%), “a minority” (11-33%), “many” (34-50%) “most” (51-90%), and “nearly all” (> 90%).

The study was approved by the Maseno University Ethics and Research Committee. All co-investigators and research staff had Human Subjects certification and had several years of experience conducting research and implementing HIV prevention programs with MSM and TW.

## Results

### Participant Characteristics

Between March 24, 2021 and August 19, 2021, 464 participants attended 25 information sessions. Of those, 444 agreed to be interviewed; 21 could not be recontacted; and 423 were interviewed – 141 from each of the three sites (Kisumu, Nairobi, Coast). Socio-demographic characteristics and reported sexual behaviors of the 423 interviewed are shown in Table I. Participants ranged in age from 18 to 35 years with a median age of 24 (IQR: 21–27). All participants were biologically male at birth. Most (59.3%) identified as gay, while 28.4% said that they were bisexual and 11.1% TW. More than half (54.4%) the participants had a secondary education or less. Most (71%) have been residents in Kisumu, Nairobi or the Coast for more than ten years. Approximately one third (32.6%) were unemployed; another 28.8% were employed with salary and the rest were either self-employed or casual laborers. Nearly half of the participants (48.5%) reported having three or more sex partners in the last three months; 71% reported having had receptive anal sex with a man in the last three months; and 13.7% reported participating in group sex (sex with more than one person at the same time). Nearly three out of five (58.6%) participants identified as sex workers, while half (51.1%) reported a condom was used by their partner the last time they engaged in receptive anal sex. Approximately two thirds (65.2%) reported having ever used daily oral PrEP and 31% reported currently taking daily oral PrEP; 9.7% were using gender affirming therapies.

### Pairwise Preferences Among all Prevention Options

A preference for an HIV prevention option was considered consistent if it was always chosen over the other options in all pairwise comparisons that included it. If an option was not consistently chosen over the other four, then “undecided” was assigned. 81% (343/443) of participants consistently expressed preference for one prevention choice over another in the pairwise choice experiment; 19% (80/423) were “undecided.” Among the five options, quarterly injection was the most frequently chosen (26%), followed closely by monthly pill (23%) and yearly implant (19%). Daily pill was by far the least preferred option (1%), followed by the condom (12%). (Table I).

Across study sites, the preference for quarterly injection was quite uniform, but other preferences were moderately variable across sites, with more participants in Kisumu preferring condoms (23.4%) and very few (4.3%) being undecided; whereas in Nairobi many more participants were undecided (29.8%) and fewer preferred implants (15.6%) and the monthly pill (16.3%). There were significant differences between those who described themselves as gay, bisexual or transexual with more TW preferring the implant (38.3%). Nearly an equal proportion of bisexual men preferred the monthly pill (31.7%) and the three-monthly injection (30.0%) and similarly for gay men (21.9% and 24.7%, respectively), but gay men were more likely to be undecided (22.7%). The main differences by level of education were among those with a primary education, more of whom were undecided (38.5%) and fewer preferred the monthly pill (15.4%) or injection (13.5%). Regarding religious affiliations, Muslims were much more likely to prefer the yearly implant compared to Catholics, Protestants and those affiliated with indigenous African religions or no religion (40.0% versus 15.3%, 17.1%, and 7.3%, respectively). There were differences according to certain reported sexual behaviors. More of those who reported one or no sex partners in the last three months (25.1%) preferred using condoms than those reporting two partners or more than three in the last three months (22.6% versus 15.2% and 4.9%), and fewer preferred the injection (15.1% versus 26.8% and 30.2%). Those who identified as sex workers (58.6%) preferred the monthly pill (27.8%) or the implant (22.6%) more than those who did not identify as sex workers. More participants who reported using a condom at last receptive sex with a man (51.1%) said that they preferred the implant (22.7%); whereas those who had not used a condom at last receptive sex preferred the monthly pill (28.4%), with equal proportions (25%) preferring the injection. There were no significant differences in preferences by age group, employment status, time of residence at each site, ever having had receptive anal sex, reporting group sex in the last three months, ever having taken PrEP, or using gender-affirming therapies.

Table II shows the number and proportion of participants who chose each option paired against each other option. All options (condoms, monthly pill, quarterly injection and yearly implant) were highly preferred (p < 0.01) over the daily pill. Condoms were preferred against the daily pill by 67% of participants and nearly half (47%) preferred condoms to the yearly implant. The monthly pill was highly preferred (p < 0.01) over the condom (78%) and the daily pill (96%) and less so over the yearly implant (66%), while the quarterly injection was the most preferred option – preferred by 72% over the condom, 96% over the daily pill, 55% over the monthly pill and 69% over the yearly implant. The yearly implant was preferred over the condom (53%) and daily pill (66%) but not over the monthly pill or the yearly injection.

### Forced-Choice Among Four PrEP Options

After being asked to provide their choices of options each paired against the other, participants were “forced” to choose the one PrEP option that they would prefer over all the others. The results are shown in Table III. As with the pairwise choices, the daily pill was seldom chosen – only 10 (2.4%) participants chose the daily pill over the other PrEP options. Slightly more participants (37.1%) chose the quarterly injection over the other options, with the monthly pill being nearly as popular (34.8%). Approximately one quarter of participants (25.8%) expressed preference for the yearly implant. The distribution of choices across sites were fairly uniform (Chi-square = 0.586) with the injection being somewhat more popular in Nairobi than elsewhere.

### Agreement Between Paired Preferences and Forced-Choice

Were participants consistent when asked to make one choice among the four PrEP options compared to when they made pairwise choices? In a way, this is a measure of participants’ commitment to one particular choice. As shown in Table IV, agreement was above 70% for the monthly pill, the quarterly injection and the yearly implant with highest agreement among those who chose the implant (87.8% agreement). Among the 51 participants who had chosen the condom among their paired choices, a few (5,9%) chose the daily pill with the rest distributed across the monthly pill (35.3%), the quarterly injection (31.4%) and the implant (23.5%). Among the 80 participants whose choices had been inconsistent during the paired preference portion of the interview (i.e., undecideds), more (41.3%) chose the monthly pill than the quarterly injection (33.8%) or the yearly implant (22.5%). The largest inconsistency between the paired choices and the forced choice was the switch from the monthly pill to the quarterly injection with 26.3% of participants switching, but 20.4% of those who had preferred the injection switched to the monthly pill. Consequently, in the final forced choice exercise, the injection was endorsed only slightly more than the monthly pill (37.1% vs. 34.8%).

### Factors Associated with the Yearly Implant Versus the Monthly Pill and Quarterly Injection

We conducted a multinomial regression of the demographic and behavioral factors associated with the choice of the monthly pill and the quarterly injection compared to the yearly implant (Table V). Daily oral pill was excluded due to few respondents expressing preference for that option. There were no differences between those who chose the yearly implant and those who chose the monthly pill or the quarterly injection by age group, level of education, study site, number of sex partners in the last three months, ever having anal sex with a man, using a condom with last receptive anal sex, or having ever taken oral PrEP. Salaried participants preferred the monthly pill and the injection over the implant significantly more than those who were unemployed. Catholics, Protestants and those who declared their religion as African indigenous or none significantly preferred the monthly pill and the quarterly injection over the yearly implant compared to Muslims. The monthly pill was preferred over the implant by those who had engaged in group sex. TW were much more likely than those who identified as gay to prefer the implant over the quarterly injection (RRR 0.33, 95% confidence interval [CI]: 0.13–0.81), as were those who were circumcised (RRR 0.39; 95% CI 0.16–0.96).

After adjustment, the monthly pill was preferred by those who were self-employed (aRRR 1.92; 95% CI 0.97–3.78), those who were Catholic (aRRR 2.86; 95% CI 1.30–6.26) or African indigenous religion or no religion (aRRR 3.06; 95% CI 0.93–10.12), and those who had engaged in group sex (aRRR 2.27; 95% CI 0.99–5.19). Those who preferred the quarterly injection over the yearly implant were more likely to be salaried (aRRR 2.31; 95% CI 1.16–4.57), and of an African indigenous religion or none (aRRR 4.01; 95% CI 1.19–13.51). Those who were circumcised (aRRR 0.47; 95% CI 0.22–1.01) and trans women (aRRR 0.38; 95% CI 0.16–0.89) were more likely to prefer the yearly implant over the quarterly injection.

### Qualitative Results – Reasons for Choices

All 423 participants responded to the question, “What are your reasons for making the (forced) choice that you did?” The most frequent responses given by the 11 participants who chose the daily pill were that one pill every day was easy to remember, and the daily pill provides the ability to start and stop at will. Two participants expressed concern that the monthly pill and the injection might have more side effects than the daily pill.

Duration of protection was the common theme across the reasons given for preferring any of the longer acting PrEP options (monthly pill, quarterly injection, yearly implant). The importance of duration was multidimensional. A longer period of protection meant a longer period during which the participant did not have to be concerned about being exposed to HIV. S/He could act more freely (e.g., have more sex partners, forget to use a condom, drink alcohol with sex) and enjoy him or herself more. Longer duration requires fewer visits to a clinic, which not only increases convenience and decreases need for costly travel, but more salient for many was reduced risk of being detected attending a health facility, equating to greater confidentiality and less stigma. Many participants mentioned that they travel frequently and either forget their daily PrEP pills or run out when they are away from their home clinic. Longer duration allows more freedom of movement.

Reasons mentioned for choosing the monthly pill specifically included: no need to carry around noisy pills, no need to store pills, ease of transition from daily to a monthly regimen, fear of pain from an injection or implant, ability to stop after just one month. A few participants expressed concern that the monthly pill may have more side effects than the daily pill, especially in the first few days of the month when there is perhaps an initial surge of medication in the blood stream.

The injection was valued for its duration of protection, but also because it was quick and easy, and less likely to be detected by others. Some participants said that they do not like pills; they have difficulty swallowing pills, and they believe that injections are “stronger and more accurate and they go directly into your blood stream.” Those who mentioned preference for the injection over the implant felt that insertion and retraction of the implant involves more pain, the implant may be detectable, and it is likely uncomfortable. Remarkably, many participants mentioned that the quarterly injection was ideal for them because they report to a health facility quarterly for their HIV test, so getting the injection at the same time would be convenient.

Nearly all of those who preferred the implant mentioned the duration of protection for a full year. “I like the implant because I can forget about protection for a whole year and I don’t need to remember my visits.” As found in the quantitative analysis, the implant is preferred by more TW than other participants, perhaps because many are sex workers whose work requires a lot of travel. Freedom to travel without having to worry about monthly or quarterly visits to a facility was considered as a significant advantage of the implant. A few of the participants who preferred the implant also mentioned that they feared injections and had difficulties swallowing pills. A minority did express concern that the implant may have more side effects and it would be difficult to extract the implant should they experience any adverse events. “The implant means a bigger commitment; you better be sure it is safe and that you want it.”

## Discussion

End-user input on early-stage design and testing of new HIV prevention approaches is critical to yielding products that achieve high uptake and adherence. Uptake and especially adherence to daily oral PrEP has proven challenging among MSM and TW in Africa; alternative regimens are needed (9–12). This study of preferences for daily oral PrEP, condoms, monthly oral pill, quarterly injection and yearly implant among MSM and TW across three sites in Kenya found nearly universal preference for longer-acting regimens over daily PrEP. Approximately 80% of participants consistently preferred one prevention option above all others. In paired choices, the daily pill was consistently and overwhelmingly eschewed by participants compared to all other possibilities. The longer acting options were preferred, but there was not one longer acting PrEP regimen that was strongly preferred over all others. The monthly pill and quarterly injections were approximately evenly preferred, with the quarterly injection only slightly preferred over the monthly pill. When “forced” to state a preference for just one choice over all others, results were similar, with 37% preferring the quarterly injection; 35% preferring the monthly pill; 25% preferring the yearly implant; and only a small minority (2%) preferring daily oral PrEP.

Our finding that longer acting alternatives will be much preferred over daily PrEP is in keeping with most studies of MSM in the United States [14, 15, 19–21], although Green et al. [15] found that among a national sample of MSM recruited online, condoms were preferred over a non-visible implant and oral PrEP. One previous investigation of PrEP choice that included MSM in South Africa, Kenya and Uganda was conducted before oral PrEP was available [22]; even then participants expressed preference for bimonthly or monthly injections over either daily or intermittent oral PrEP. Two other studies, both conducted in Cape Town, South Africa, were discreet choice experiments focusing not on specific products, but on PrEP product attributes including the form of delivery, duration, insertion location, soreness, and delivery facility [22, 24]. Broadly consistent with our findings among Kenyan MSM, duration of protection was the most salient product attribute with injectables preferred over implants.

The yearly implant was preferred by approximately one quarter of participants in our study. Notably, more TW (38%) regarded an implant as preferable than did men who identified as gay (17%) or bisexual (18%), and TW were significantly more likely to prefer the implant over the quarterly injection in adjusted analyses. Responding to qualitative questioning about the reasons for their choices, some TW said that an implant would make them feel more feminine; others felt that the implant could be combined with hormonal therapies; and others felt that the protection offered by an implant would be more secure while also offering longer intervals between clinic visits. Reasons for not preferring the implant were that it involved an invasive surgical procedure, that it might be detectable, that it probably had more side effects because it had to last so long, and that it would be more difficult to discontinue PrEP should a person change their mind or should they experience adverse events. Clearly, it will be crucial that potential users of the implant, as with any of the PrEP products, receive sufficient educational counseling to ensure that they fully understand the benefits and risks of adopting the product and the prevention options available to them so that they can make informed PrEP decisions.

While a significant proportion of TW expressed preference for the yearly implant, it is important to note that it was not a majority. This was a finding consistent across all participants irrespective of demographic or behavioral characteristic: no one product was preferred by most, not to mention all, individuals within a group or geographic area. As has been stressed by others, a diversity of PrEP products will need to be developed and promoted to reflect variation in preferences [20, 23, 25, 26]. Moreover, as revealed by responses to our qualitative questioning, every product has its perceived advantages and disadvantages. Adoption or rejection of any one will be the result of weighing trade-offs. Delivery of accurate information by providers with messages based on full understanding of potential concerns will be critical for product adoption and adherence.

When we examined the demographic and behavioral characteristics associated with the three different long-acting options, those who were Catholic and those who reported engaging in group sex were more likely to prefer the monthly pill. Those whose employment was salaried and whose religious affiliation was with an African indigenous church or no religion were more likely to prefer the quarterly injection. The reasons behind these preferences are not clear and deserve exploration through further qualitative work. Possible reasons for those with salaried employment to prefer the quarterly injection are that the injection requires fewer visits to a clinic which could take them away from their work, and that an injection is not detectable by others, which could be an important consideration for persons who interact with the same co-workers on a long-term daily basis making it more likely that an implant would eventually be detected. In addition to significantly more TW expressing preference for the yearly implant compared to MSM, more circumcised compared to uncircumcised participants chose the implant as their preferred option. Since 91 – 95% of males over age 15 years in Kenya are circumcised [26], further exploration of the reasons for the implant being a more attractive choice to them could be helpful for developing education and promotion interventions.

### Limitations

Our results are from a sample of MSM and TW across just three urban and peri-urban sites in Kenya and thus may not be generalizable to other areas or other key populations in Kenya or in other areas of sub-Saharan Africa. Also, our results are from responses to questions about hypothetical products and scenarios. The preferences of MSM and TW may well change substantially once specific products are offered and the requirements for implementation are established. Observational studies will be necessary to assess whether and how preferences change over time as new products are proven efficacious and are made available. Methods from implementation science will have to be deployed to evaluate different models of product introduction, access, and ongoing delivery to achieve optimal uptake and adherence. Understanding variation in preferences will be critical to that effort.

In this study we chose not to include information about the efficacy of the long-acting options and told participants that all the products would confer efficacy equal to daily oral FTC/TDF. This appears to be the case with the recently tested bi-monthly cabotagrivir injection [28], and early trials of long-acting islatravir suggest similarly high levels of efficacy [29, 30]. Other studies of end user preferences for PrEP have focused less on specific products and regimens and more on the preferences for potential product attributes such as dosing frequency, duration of protection, method of delivery, site of insertion or injection, pain levels, access and detectability. [21, 22, 24] These studies are useful for understanding tradeoffs between attributes and for developing products that will optimize acceptability, uptake and adherence. This study was intended to assess preferences for specific products that are already under development and in early and late stage trials [28, 29], rather than general attributes. Nevertheless, our findings are broadly consistent with the findings of previous studies indicating strong preferences for longer-acting products compared to daily oral PrEP and for injectables over implants ([23, 25]. In addition, the results of this and other studies strongly indicate that not just one product is likely to be preferable over all others.

## Conclusion

Introduction of daily PrEP has had very limited success in sub-Saharan Africa in general and among MSM and TW especially. Our results reflect the lack of enthusiasm for daily PrEP and strong preference among MSM and TW for longer-acting alternatives. Whether across pairwise comparisons or when “forced” to choose one regimen over all other options, fewer than 3% chose daily PrEP. Monthly pills and quarterly injections were approximately equally preferred; however, yearly implants were also preferable among approximately one quarter of participants. Importantly, no one product was highly preferred over all others. To achieve the rates of PrEP uptake and adherence necessary for protecting large proportions of vulnerable MSM and TW, a variety of long-acting products should be developed and made accessible to appeal to a diversity of preferences [20, 26].

## Figures and Tables

**Table I:** Characteristics of 423 MSM and TW study participants by paired choice of HIV prevention options^1^

Variable	Total N (%)	Condom n = 51 (12%) n (%)	Daily pill n = 3 (1%) n (%)	Implant n = 82 (19%) n (%)	Monthly Pill n = 99 (23%) n (%)	Injection n = 108 (26%) n (%)	Undecided n = 80 (19%) n (%)	p-value^¥^
Site								**< 0.001**
Kisumu	141(33.3)	33(23.4)	2(1.4)	30(21.3)	36(25.5)	34(24.1)	6(4.3)	
Nairobi	141(33.3)	17(12.1)	0(0.0)	22(15.6)	23(16.3)	37(26.2)	42(29.8)	
Coast	141(33.3)	1(0.7)	1(0.7)	30(21.3)	40(28.4)	37(26.2)	32(22.7)	
Which of the following best describes you?								**0.006**
Gay	251(59.3)	33(13.1)	2(0.8)	42(16.7)	55(21.9)	62(24.7)	57(22.7)	
Bisexual	120(28.4)	12(10.0)	1(0.8)	21(17.5)	38(31.7)	36(30.0)	12(10.0)	
Transgender woman	47(11.1)	6(12.8)	0(0.0)	18(38.3)	6(12.8)	9(19.1)	8(17.0)	
Unsure/Questioning/other	5(1.2)	0(0.0)	0(0.0)	1(20.0)	0(0.0)	1(20.0)	3(60.0	
Age group (years)								**0.260**
18–24	248(58.6)	25(10.1)	1(0.4)	41(16.5)	66(26.6)	64(25.8)	51(20.6)	
25–29	111(26.2)	20(18.0)	1(0.9)	27(24.3)	19(17.1)	27(24.3)	17(15.3)	
30 +	64(15.1)	6(9.4)	1(1.6)	14(21.9)	14(21.9)	17(26.6)	12(18.8)	
Highest level of education								**0.010**
Primary	52(12.3)	2(3.8)	1(1.9)	14(26.9)	8(15.4)	7(13.5)	20(38.5)	
Secondary	178(42.1)	21(11.8)	2(1.1)	33(18.5)	44(24.7)	46(25.8)	32(18.0)	
Tertiary	112(26.5)	19(17.0)	0(0.0)	17(15.2)	31(27.7)	31(27.7)	14(12.5)	
University	80(18.9)	9(11.3)	0(0.0)	17(21.3)	16(20.0)	24(30.0)	14(17.5)	
Employment status								**0.301**
Unemployed	138(32.6)	19(13.8)	1(0.7)	29(21.0)	27(19.6)	33(23.9)	29(21.0)	
Salaried	122(28.8)	12(9.8)	0(0.0)	17(13.9)	30(24.6)	37(30.3)	26(21.3)	
Self employed	115(27.2)	13(11.3)	1(0.9)	25(21.7)	35(30.4)	22(19.1)	19(16.5)	
Casual/other	48(11.3)	7(14.6)	1(2.1)	11(22.9)	7(14.6)	16(33.3)	6(12.5)	
How long have you lived in Kisumu/Nairobi/Coast								**0.367**
<5yrs	67(15.8)	4(6.0)	0(0.0)	11(16.4)	17(25.4)	23(34.3)	12(17.9)	
5-10yrs	56(13.2)	9(16.1)	1(1.8)	9(16.1)	17(30.4)	9(16.1)	11(19.6)	
<10yrs	300(70.9)	38(12.7)	2(0.7)	62(20.7)	65(21.7)	76(25.3)	57(19.0)	
Religious affiliation								**0.004**
Muslim	70(16.5)	2(2.9)	0(0.0)	28(40.0)	12(17.1)	15(21.4)	13(18.6)	
Catholic	137(32.4)	20(14.6)	2(1.5)	21(15.3)	32(23.4)	33(24.1)	29(21.2)	
Protestant	175(41.4)	21(12.0)	1(0.6)	30(17.1)	45(25.7)	49(28.0)	29(16.6)	
African/no religion	41(9.7)	8(19.5)	0(0.0)	3(7.3)	10(24.4)	11(26.8)	9(22.0)	
Circumcised								**0.001**
No	68(16.1)	6(8.8)	2(2.9)	19(27.9)	9(13.2)	12(17.6)	20(29.4)	
Yes	355(83.9)	45(12.7)	1(0.3)	63(17.7)	90(25.4)	96(27.0)	60(16.9)	
Number of male sex partners in the last 3 months?								**0.001**
<=1	106(25.1)	24(22.6)	1(0.9)	17(16.0)	28(26.4)	16(15.1)	20(18.9)	
two	112(26.5)	17(15.2)	1(0.9)	23(20.5)	21(18.8)	30(26.8)	20(17.9)	
3 +	205(48.5)	10(4.9)	1(0.5)	42(20.5)	50(24.4)	62(30.2)	40(19.5)	
Identify as sex worker								**0.013**
No	175(41.4)	28(16.0)	2(1.1)	26(14.9)	30(17.1)	46(26.3)	43(24.6)	
Yes	248(58.6)	23(9.3)	1(0.4)	56(22.6)	69(27.8)	62(25.0)	37(14.9)	
Ever had receptive anal sex with a man								**0.430**
No	122(28.8)	17(13.9)	0(0.0)	21(17.2)	35(28.7)	28(23.0)	21(17.2)	
Yes	301(71.2)	34(11.3)	3(1.0)	61(20.3)	64(21.3)	80(26.6)	59(19.6)	
Condom use at last incertive sex with a man								**0.239**
No	126(29.8)	10(7.9)	2(1.6)	24(19.0)	28(22.2)	37(29.4)	25(19.8)	
Yes	290(68.6)	41(14.1)	1(0.3)	51(17.6)	71(24.5)	71(24.5)	55(19.0)	
Condom use at last receptive sex with a man								**0.091**
No	201(47.5)	19(9.5)	2(1.0)	32(15.9)	57(28.4)	51(25.4)	40(19.9)	
Yes	216(51.1)	32(14.8)	1(0.5)	49(22.7)	41(19.0)	55(25.5)	38(17.6)	
Group sex in past 3 months								**0.220**
No	361(85.3)	49(13.6)	3(0.8)	71(19.7)	85(23.5)	92(25.5)	61(16.9)	
Yes Ever taken PrEP	58(13.7)	2(3.4)	0(0.0)	11(19.0)	14(24.1)	16(27.6)	15(25.9)	**0.243**
No	147(34.8)	23(15.6)	1(0.7)	24(16.3)	32(21.8)	33(22.4)	34(23.1)	
Yes	276(65.2)	28(10.1)	2(0.7)	58(21.0)	67(24.3)	75(27.2)	46(16.7)	
Using any gender affirming therapies								**0.530**
No	382(90.3)	46(12.0)	3(0.8)	70(18.3)	93(24.3)	98(25.7)	72(18.8)	
Yes	41(9.7)	5(12.2)	0(0.0)	12(29.3)	6(14.6)	10(24.4)	8(19.5)	

1 Columns with total N (%) are column percents. All other values are row percents

¥ Pearson’s Chi-square test of independence /Fisher’s Exact test

**Table II:** Number and proportion of participants choosing each HIV prevention option paired against each other option (row to column in each paired choice) N = 423^1^

Variables	Condom	Daily Pill	Monthly Pill	Quarterly Injection	Annual Implant
Condom		274/412 (67%)*	94/423 (22%)	117/423 (28%)	188/402 (47%)
Daily pill	138/412 (34%)	15/423 (4%)	55/423 (13%)	142/423 (34%)
Monthly pill	329/423 (78%)*	408/423 (96%)*		192/423 (45%)	262/395 (66%)*
Quarterly Injection	306/423 (72%)*	368/423 (87%)*	231/423 (55%)		281/410 (69%)*
Yearly Implant	214/402 (53%)	281/423 (66%)*	133/395 (34%)	129/410 (31%)	

1 N <423 in some cells due to participants declining to make a choice

* Significant preference of row option over column option at p < 0.01 by Dunn’s Pairwise Test analysis following Kruskal-Wallis test

**Table III:** Forced choice preferences among four PrEP options by 423 MSM and TG participants at each of three study sites

Variables	Total(N)	Kisumu (n = 141) n (%)	Nairobi (n = 141) n (%)	Coast (n = 141) n (%)	p-value^¥^
Choose one option among four					0.586
Option 1: Daily Pill	10(2.4)	4(2.8)	3(2.1)	3(2.1)	
Option 2: Monthly Pill	147(34.8)	52(36.9)	40(28.4)	55(39.0)	
Option 3: Quarterly Injection	157(37.1)	51(36.2)	59(41.8)	47(33.3)	
Option 4: Yearly Implant	109(25.8)	34(24.1)	39(27.7)	36(25.5)

¥ Pearson’s Chi-square test of independence /Fisher’s Exact test

**Table IV:** Agreement between participants’ paired HIV prevention preferences and their forced choices^1^

Paired preference	Daily pill	Monthly pill	Quarterly Injection	Yearly Implant	Total
	10 (2.4%)	147 (34.8%)	157 (37.1%)	109 (25.8%)	N = 423
Undecided	2(2.5)	33 (41.3)	27 (33.8)	18 (22.5)	80 (18.9%)
Condom	5 (9.8)	18 (35.3)	16 (31.4)	12 (23.5)	51 (12.1%)
Daily pill	2 (66.7)	0	1 (33.3)	0	3 (0.7%)
Yearly Implant	0	3 (3.7)	7 (8.5)	72 (87.8)	82 (19.4%)
Monthly pill	1 (1.0)	71 (71.7)	26 (26.3)	1 (1.0)	99 (23.4%)
Quarterly Injection	0	22 (20.4)	80 (74.1)	6 (5.7)	108 (25.5%)

1 Paired preference options are shown in the first column. The total number and percent that chose each option during the paired choice portion of the interview is in the last column. Other columns show the number and percent who chose a particular preference when “forced” to choose one among the daily pill, monthly pill, quarterly injection and yearly implant. The numbers and percents in the columns reflect the fidelity of participants’ choices going from paired choice to forced choice. For example, among the 99 participants who consistently preferred the monthly pill when presented with pairwise options, 71 (71.7%) chose the monthly pill over all other options when forced to choose just one option among all; whereas 28 (28.3%) chose a different option

**Table V:** Multinomial regression analyses of factors associated with forced choice of the annual implant versus the monthly pill and the quarterly injection

	Univariate		Multivariable Adjusted	
	Monthly pill vs. Implant Unadjusted RRR 1(95% CI), p-value	Quarterly Injection vs. Implant Unadjusted RRR 1 (95% CI), p-value	Monthly pill vs. Implant Adjusted RRR 1 (95% CI), p-value^2^	Quarterly Injection vs. Implant Adjusted RRR 1 (95% CI), p-value^2^
Age (years)				
18–24	Reference	Reference		
25–29	0.94 (0.24–1.90), 0.88	1.36 (0.70–2.66), 0.36		
30 and older	1.14 (0.47–2.76), 0.77	0.76 (0.31–1.88), 0.55		
Education Completed				
Primary	Reference	Reference		
Secondary	0.99 (0.38–2.58), 0.98	1.17 (0.43–3.13), 0.76		
Tertiary	0.96 (0.34–2.69), 0.93	1.30 (0.46–3.17), 0.62		
University	1.09 (0.35–3.40), 0.88	1.23 (0.40–3.84), 0.72		
Employment Status				
Unemployed	Reference	Reference	Reference	Reference
Salaried	2.11 (0.99–4.50), 0.05	2.35 (1.16–4.79), 0.18	1.87 (0.91–3.84), 0.09	2.31 (1.16–4.57), 0.02
Self-employed	1.86 (0.89–3.87), 0.09	1.92 (0.97–3.78), 0.06	1.06 (0.51–2.20), 0.88	1.18 (0.60–2.34), 0.63
Casual/Other	0.59 (0.22–1.61), 0.30	1.92 (0.97–3.78), 0.06	0.87 (0.35–2.15), 0.76	0.96 (0.41–2.27), 0.93
Religion				
Muslim	Reference	Reference	Reference	Reference
Catholic	3.87 (1.62–9.28), 0.10	1.66 (0.71–3.87), 0.24	2.86 (1.30–6.26), 0.01	1.56 (0.71–3.33), 0.27
Protestant	2.81 (1.20–6.54), 0.02	2.41 (1.09–5.33), 0.03	1.33 (0.71–2.50), 0.37	1.36 (0.73–2.52), 0.33
African/none	10.2 (2.7–38.9), 0.01	7.08 (1.88–26.7), 0.01	3.06 (0.93–10.1), 0.07	4.01 (1.19–13.5), 0.03
Study Site				
Kisumu	Reference	Reference		
Nairobi	0.57 (0.27–1.18), 0.31	0.81 (0.40–1.64), 0.56		
Coast	1.67 (0.70–3.99), 0.25	1.16 (0.49–2.71), 0.74		
Self Description^3^				
Gay	Reference	Reference	Reference	Reference
Bisexual	1.31 (0.69–2.49), 0.41	0.88 (0.47–1.65), 0.68	1.57 (0.86–2.88), 0.14	0.94 (0.52–1.72). 0.84
Trans Woman	0.50 (0.21–1.18), 0.11	0.33 (0.13–0.81), 0.02	0.62 (0.27–1.41), 0.25	0.38 (0.16–0.89), 0.03
Unsure/Questioning	2.98 (0.22–40.4), 0.41	1.14 (0.06–22.2), 0.93	3.48 (0.30–40.5), 0.31	1.05 (0.06–19.1), 0.97
Number Sex Partners^4^				
0–1	Reference	Reference		
Two	0.75 (0.36–1.59), 0.46	0.86 (0.41–1.81), 0.69		
Three or more	0.62 (0.28–1.35), 0.23	1.09 (0.51–2.33), 0.82		
Receptive Anal Sex				
No	Reference	Reference		
Yes	1.44 (0.68–3.03), 0.34	1.26 (0.61–2.61), 0.53		
Used Condom Last Sex				
No	Reference	Reference		
Yes	0.87 (0.44–1.68), 0.69	0.70 (0.36–1.33), 0.27		
Circumcised				
No	Reference	Reference	Reference	Reference
Yes	0.33 (0.14–0.81), 0.02	0.39 (0.16–0.96). 0.04	0.57 (0.27–1.18), 0.13	0.47 (0.22–1.01), 0.05
Group Sex				
No	Reference	Reference	Reference	Reference
Yes	2.45 (1.03–5.08), 0.04	1.50 (0.62–3.59), 0.37	2.27 (0.99–5.19), 0.05	1.61 (0.69–3.75), 0.27
Ever taken oral PrEP				
No	Reference	Reference		
Yes	0.95 (0.51–1.75), 0.86	1.10 (0.61–2.01), 0.75		

^1^ RRR = Relative Risk Ratio; ^2^ Multinomial Logistic Regression; ^3^ Response to the question, “Which of the following best describes you?”; ^4^ Number of sex partners during the last three months.

## Data Availability

The *data* that support the findings of this study are *available* from the corresponding author, RCB, *upon reasonable request*.
